# An *ABCA4* loss-of-function mutation causes a canine form of Stargardt disease

**DOI:** 10.1371/journal.pgen.1007873

**Published:** 2019-03-19

**Authors:** Suvi Mäkeläinen, Marta Gòdia, Minas Hellsand, Agnese Viluma, Daniela Hahn, Karim Makdoumi, Caroline J. Zeiss, Cathryn Mellersh, Sally L. Ricketts, Kristina Narfström, Finn Hallböök, Björn Ekesten, Göran Andersson, Tomas F. Bergström

**Affiliations:** 1 Department of Animal Breeding and Genetics, Swedish University of Agricultural Sciences, Uppsala, Sweden; 2 Department of Neuroscience, Uppsala University, Uppsala, Sweden; 3 Department of Ophthalmology, Faculty of Medicine and Health, Örebro University, Örebro, Sweden; 4 Yale University School of Medicine, New Haven, Connecticut, United States of America; 5 Kennel Club Genetics Centre, Animal Health Trust, Lanwades Park, Kentford, Newmarket, Suffolk, United Kingdom; 6 Section for Comparative Ophthalmology, College of Veterinary Medicine, University of Missouri-Columbia, Missouri, United States of America; 7 Department of Clinical Sciences, Swedish University of Agricultural Sciences, Uppsala, Sweden; Stanford University School of Medicine, UNITED STATES

## Abstract

Autosomal recessive retinal degenerative diseases cause visual impairment and blindness in both humans and dogs. Currently, no standard treatment is available, but pioneering gene therapy-based canine models have been instrumental for clinical trials in humans. To study a novel form of retinal degeneration in Labrador retriever dogs with clinical signs indicating cone and rod degeneration, we used whole-genome sequencing of an affected sib-pair and their unaffected parents. A frameshift insertion in the ATP binding cassette subfamily A member 4 (*ABCA4*) gene (c.4176insC), leading to a premature stop codon in exon 28 (p.F1393Lfs*1395), was identified. In contrast to unaffected dogs, no full-length ABCA4 protein was detected in the retina of an affected dog. The *ABCA4* gene encodes a membrane transporter protein localized in the outer segments of rod and cone photoreceptors. In humans, the *ABCA4* gene is associated with Stargardt disease (STGD), an autosomal recessive retinal degeneration leading to central visual impairment. A hallmark of STGD is the accumulation of lipofuscin deposits in the retinal pigment epithelium (RPE). The discovery of a canine homozygous *ABCA4* loss-of-function mutation may advance the development of dog as a large animal model for human STGD.

## Author summary

Stargardt disease (STGD) is the most common inherited retinal disease causing visual impairment and blindness in children and young adults, affecting 1 in 8–10 thousand people. For other inherited retinal diseases, the dog has become an established comparative animal model, both for identifying the underlying genetic causes and for developing new treatment methods. To date, there is no standard treatment for STGD and the only available animal model to study the disease is the mouse. As a nocturnal animal, the morphology of the mouse eye differs from humans and therefore the mouse model is not ideal for developing methods for treatment. We have studied a novel form of retinal degeneration in Labrador retriever dogs showing clinical signs similar to human STGD. To investigate the genetic cause of the disease, we used whole-genome sequencing of a family quartet including two affected offspring and their unaffected parents. This led to the identification of a loss-of-function mutation in the *ABCA4* gene. The findings of this study may enable the development of a canine model for human STGD.

## Introduction

Inherited retinal dystrophies are a genetically and clinically heterogeneous group of eye diseases leading to severe visual impairment in both humans and dogs [1–6]. These diseases include various forms of retinitis pigmentosa (RP), Leber congenital amaurosis (LCA), age-related macular degeneration (AMD), cone-rod dystrophies (CRD), and Stargardt disease (STGD) and are caused by many different mutations leading to deterioration of neuroretinal and retinal pigment epithelial (RPE) function. Over 100 years ago, progressive retinal atrophy (PRA) was described as a canine equivalent of human RP [7] and is today the most common inherited retinal degenerative disease in dogs [8]. The shared phenotypic similarity of inherited retinal dystrophies in dogs and humans has made canine models attractive for gene discovery and for experimental treatments, including gene therapy [6, 9–13]. The development of gene therapy for *RPE65*-mediated LCA is an example where a canine comparative model has been instrumental for proof-of-principle trials [9, 11, 14–16]. The identification of the p.C2Y mutation (OMIM: 610598.0001) in the *PRCD* gene is another illustrative example of the benefits of using canine genetics to find homologous candidate genes for human retinal dystrophies; the *PRCD* gene was initially mapped and identified in PRA-affected dogs and subsequently in a human family with RP [17]. This mutation segregates in multiple dog breeds, including the Labrador retriever, where no other causative genetic variants for inherited retinal degenerations have been identified. In this study, a Labrador retriever sib-pair, one male and one female, negative for the p.C2Y mutation, was diagnosed with a form of retinal disease which until now had not been characterized clinically. To identify genetic variants associated with this novel canine retinal disease, we performed whole-genome sequencing (WGS) of the two affected individuals and their unaffected parents.

## Results and discussion

**Fig 1:**
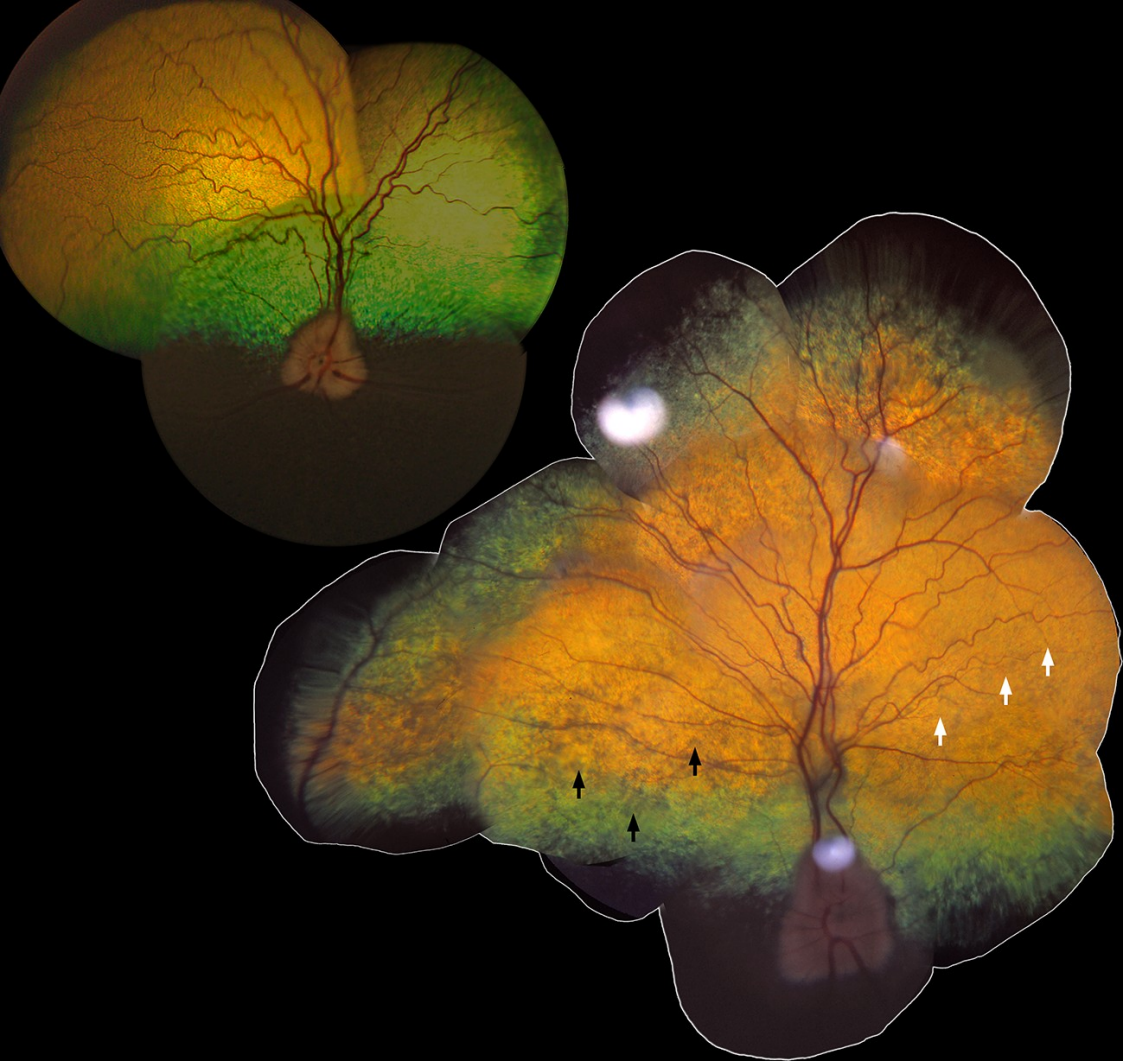
Retinal morphology *in vivo* in canine Stargardt disease. The tapetal fundus of the right eye from an 11-year-old unaffected Labrador retriever (upper left; LAB27) and a 10-year-old affected dog (lower right; LAB4). Black arrows show areas with abnormal, grayish, hyporeflective appearance and white arrows indicate attenuation of the retinal blood vessels.

The affected sib-pair (LAB3 and LAB4, see **S1 Fig**) was visually impaired under both daylight and dimlight conditions when examined at 10 years of age. Their pupils were dilated under daylight conditions and pupillary light and dazzle reflexes were abnormal, whereas menace responses were present. On indirect ophthalmoscopy, the tapetal reflectivity varied between normal to grayish hyporeflection when the indirect ophthalmoscopy lens was tilted slightly back and forth, both in the visual streak, as well as in the more peripheral parts of the tapetal fundus in both eyes of the affected dogs. The visual streak is an area of high photoreceptor cell density in the canine retina, located superior to the optic disc and extending horizontally from the nasal to the temporal region [18]. Furthermore, a mild to moderate vascular attenuation was observed, as seen in the fundus photograph, taken at the age of 10 years, of the affected male (LAB4) and compared to a fundus photograph of an unaffected, age-matched Labrador retriever dog (LAB27) (**Fig 1**). These ophthalmoscopic findings were symmetrical between the eyes of the affected dogs, diffusely spread over the tapetal fundus and not strictly confined to the visual streak or area centralis.

The WGS of the family quartet (LAB1, LAB2, LAB3 and LAB4, see **S1 Fig**) resulted in an average coverage of 18.2x (**S1 Table**) and the identification of 6.0 x 10^6^ single nucleotide variants (SNVs) and 1.9 x 10^6^ insertions/deletions (INDELs), of which 48,299 SNVs and 5,289 INDELs were exonic. We used conditional filtering to identify 322 SNVs (of which 117 were nonsynonymous) and 21 INDELs that were consistent with an autosomal recessive pattern of inheritance (**S2 Table**). To further reduce the number of candidate variants, we compared the positions of the variants to 23 additional dog genome sequences to identify 18 nonsynonymous SNVs in 13 different genes and four INDELs in four genes that were private to the Labrador retriever family (**S2 and S3 Tables**). Fourteen of these genes were not strong candidates based on reported function and predicted effect and were not considered further. The remaining three genes, *KIAA1549*, Usherin *(USH2A)*, and ATP binding cassette subfamily A member 4 (*ABCA4)* are listed in the Retinal Information Network (RetNet) database as associated with human retinal diseases and thus considered as causative candidates for canine retinal degeneration [19]. However, the variant in the *KIAA1549* gene was predicted to have a neutral effect on the protein structure (PROVEAN score -2.333, Polyphen-2 score 0.065) and was therefore discarded. The genetic variants in the *USH2A* (exon 43; c.7244C>T) and *ABCA4* (exon 28; c.4176insC) genes were validated by Sanger sequencing. Mutations in the human *USH2A* gene are associated with Usher syndrome and RP, resulting in hearing loss and visual impairment [20]. The identified nonsynonymous substitution in the *USH2A* gene was scored as “probably damaging” using Polyphen-2 (score of 0.97) and as “deleterious” using PROVEAN (score of -4.933) (**S3 Table**). The insertion in the *ABCA4* gene was predicted to result in a premature stop-codon at amino acid position 1395. Next, we evaluated if the genetic variants of *USH2A* and *ABCA4* were concordant with the disease by genotyping eight additional clinically affected and fourteen unaffected Labrador retrievers. Out of these 22 dogs, 16 were related to the family quartet used in the WGS (**S1 Fig**). The *USH2A* variant was discordant with the disease phenotype and was therefore excluded from further analysis (**S4 Table**). In contrast, all eight affected individuals were homozygous for the *ABCA4* insertion and the 14 unaffected individuals were either heterozygous or homozygous for the wild-type allele (**S4 Table**).

**Fig 2:**
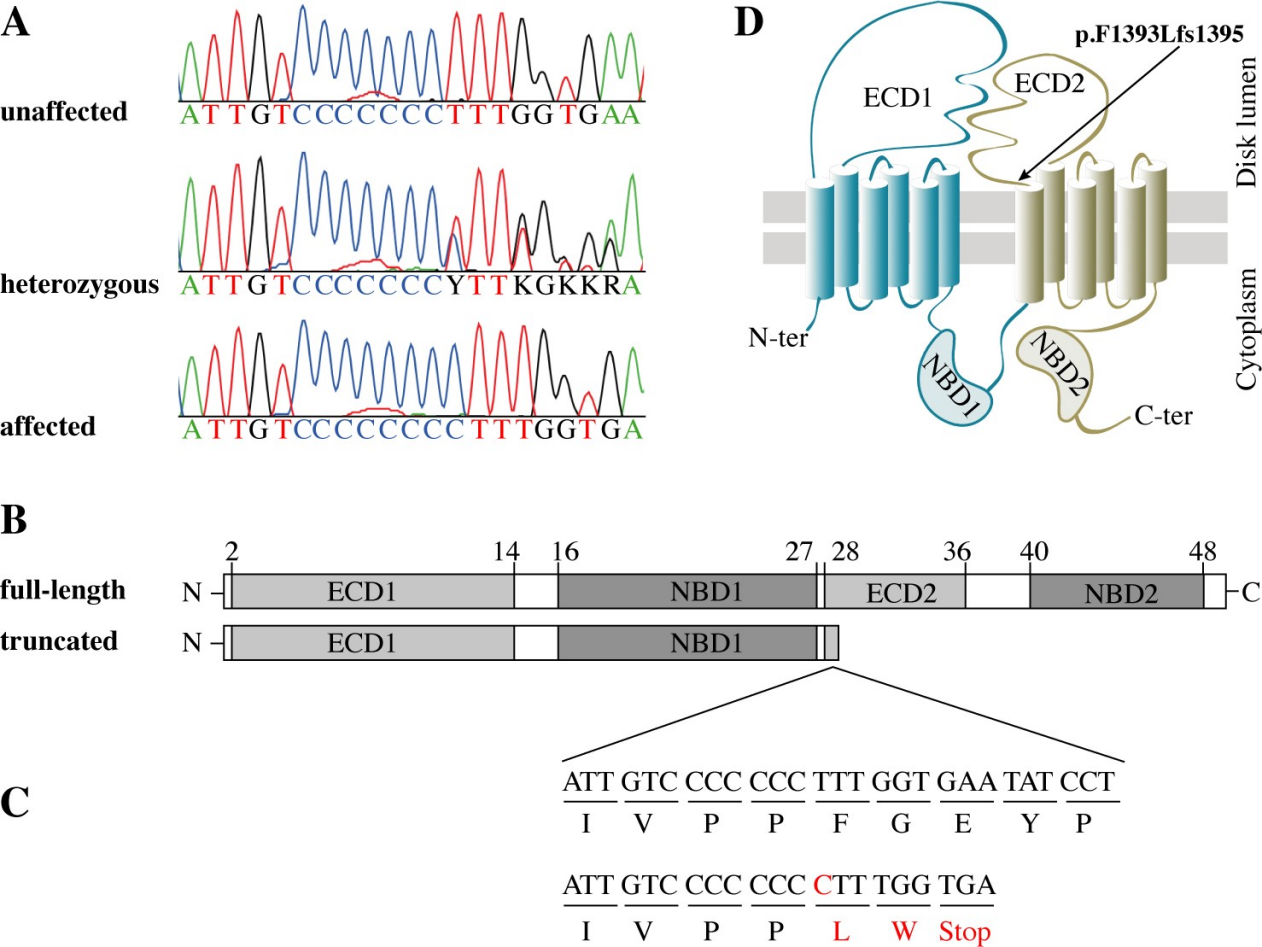
Loss-of-function mutation in the canine *ABCA4* gene. **(A)** Sanger sequencing traces spanning positions Chr6:55,146,545–55,146,564 (CanFam3.1) in exon 28 of the *ABCA4* gene of a wild-type, unaffected (*ABCA4*^+/+^) dog, a heterozygous (*ABCA4*^+/-^) dog, and a homozygous (*ABCA4*^-/-^) affected dog. **(B)** Predicted domain structure of canine full-length ABCA4 protein, based on the proposed human structure [28], and the putative truncated product as a result of the premature stop codon at amino acid residue 1,395. The inferred canine exon numbers are indicated. **(C)** Schematic representation of the region where the insertion of cytosine (C) is found showing the nucleotide and amino acid sequences of a full-length (top) and truncated (bottom) protein. **(D)** Predicted topological organization of ABCA4 [29, 30] with the insertion leading to a premature stop codon marked with an arrow. ECD1 = first extracellular domain; TMD1 = first membrane-spanning region; NBD1 = first nucleotide-binding domain; ECD2 = second extracellular domain; TMD2 = second membrane-spanning region; NBD2 = second nucleotide-binding domain.

The identified variant in the *ABCA4* gene is a single base pair (bp) insertion of a cytosine (C) in a cytosine mononucleotide-repeat region in exon 28, where the canine reference sequence consists of seven cytosines (CanFam3.1 Chr6:55,146,550–55,146,556) (**Fig 2A**). The single bp insertion in this region results in a non-synonymous substitution at the first codon downstream of the repeat, and subsequently leads to a premature stop codon (p.F1393Lfs*1395) (**Fig 2C**). If translated, this would result in a truncation of the last 874 amino acid residues of the wild-type ABCA4 protein (**Fig 2B and 2C**). Both the human and the dog *ABCA4* gene consists of 50 exons and encodes a ~250 kDa ABC transporter protein (**Fig 2D**) (human and dog ABCA4 consists of 2,273 and 2,268 amino acid residues, respectively) [21–23]. ABCA4 is a flippase, localized to the disc membranes of photoreceptor outer segments and facilitates the clearance of all-*trans*-retinal from the photoreceptor discs [24–26].

**Fig 3:**
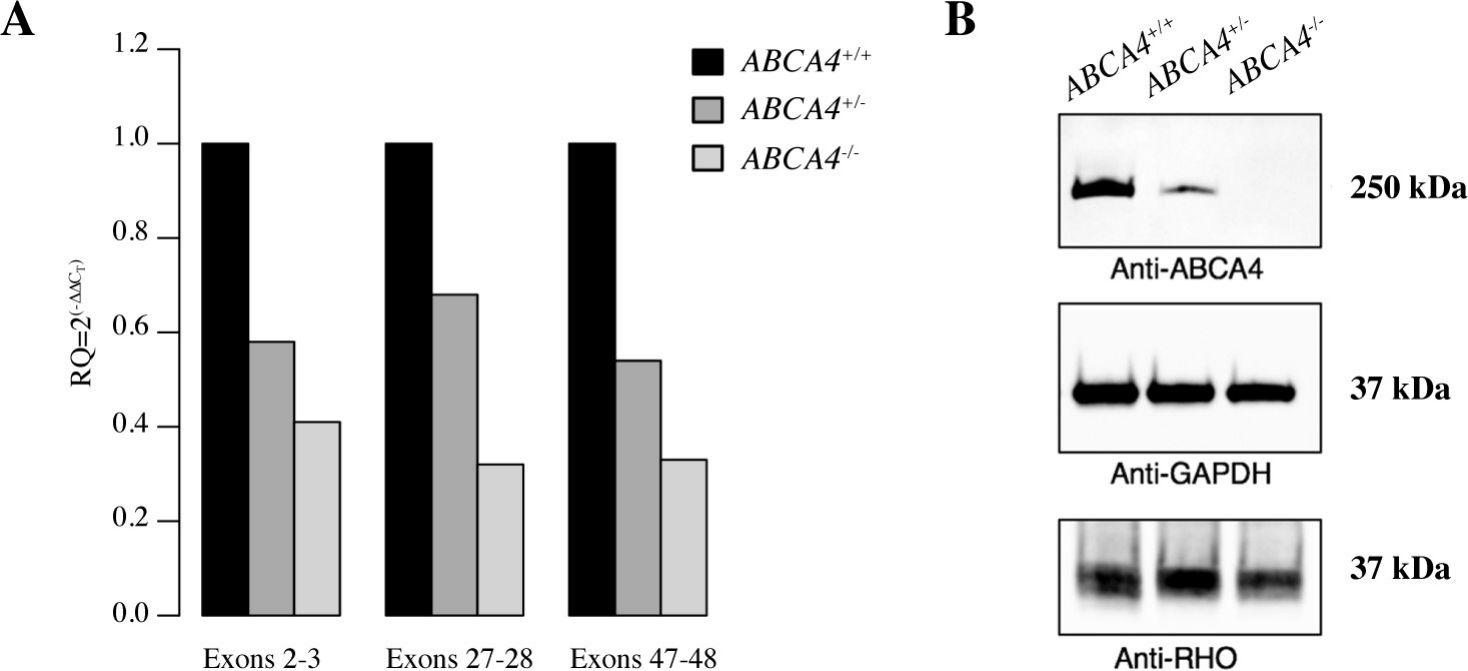
Characterization of *ABCA4* mRNA expression and western blot analyses of ABCA4 protein levels in the canine retina. **(A)** Relative *ABCA4* mRNA expression levels by quantitative RT-PCR in three different regions in three dogs with different genotypes (*ABCA4*^+/+^, *ABCA4*^+/-^, and *ABCA4*^-/-^), normalized to *GAPDH* expression. **(B)** Western blot analyses of ABCA4 (above), GAPDH (middle), and RHO (below) protein levels in retinal tissue of dogs with the three different genotypes.

To compare retinal *ABCA4* gene expression in the affected male (LAB4), his heterozygous sibling (LAB6), and a wild-type Labrador retriever (LAB24), we performed quantitative RT-PCR (qPCR). Primers were designed to amplify three different regions of the gene. The amplicons spanned the 5´-end (exons 2–3), the identified insertion (exons 27–28) and the 3´-end of the *ABCA4* gene (exons 47–48) (**S5 Table**). Each of the three primer pairs amplified a product of expected size in all three individuals. This suggests that despite the insertion leading to a premature stop codon in exon 28, the transcripts are correctly spliced. Relative levels of *ABCA4* mRNA were lower for the allele with the insertion in comparison to the wild-type allele (**Fig 3A**). This is consistent with nonsense-mediated decay (NMD) degrading a fraction of the transcripts with premature translation stop codon [27]. Transcripts not targeted by NMD could potentially be translated into a truncated protein of only 1,394 amino acid residues including the first extracellular domain (ECD1) and the first nucleotide-binding domain (NBD1) (**Fig 2B**) but lacking most of the second extracellular domain (ECD2) and the second nucleotide-binding domain (NBD2) [28–30] (**Fig 2B–2D**). The NBDs are conserved across species and the NBD2, which is also referred to as the ATP binding cassette of the ABCA4 protein, has been shown to be particularly critical for its function as a flippase [28, 30].

To investigate the presence of full-length protein, we performed western blot analysis using an anti-ABCA4 antibody recognizing a C-terminal epitope and detecting a protein product with an approximate size of ~250 kDa. We observed a single, correctly-sized band in samples prepared from both wild-type (LAB24) and heterozygous (LAB6) dogs. The intensity of staining in retinal protein samples from the heterozygous individual was markedly lower in comparison to the samples from the wild-type retina (**Fig 3B**). In contrast, no band was detected in the retinal sample from the affected dog (LAB4). To confirm the presence of photoreceptor cells, we used an anti-RHO antibody and detected rhodopsin in all three samples (**Fig 3B**). These results suggest that no full-length ABCA4 protein product is produced as a result of the insertion leading to a frameshift and a premature stop codon.

**Fig 4:**
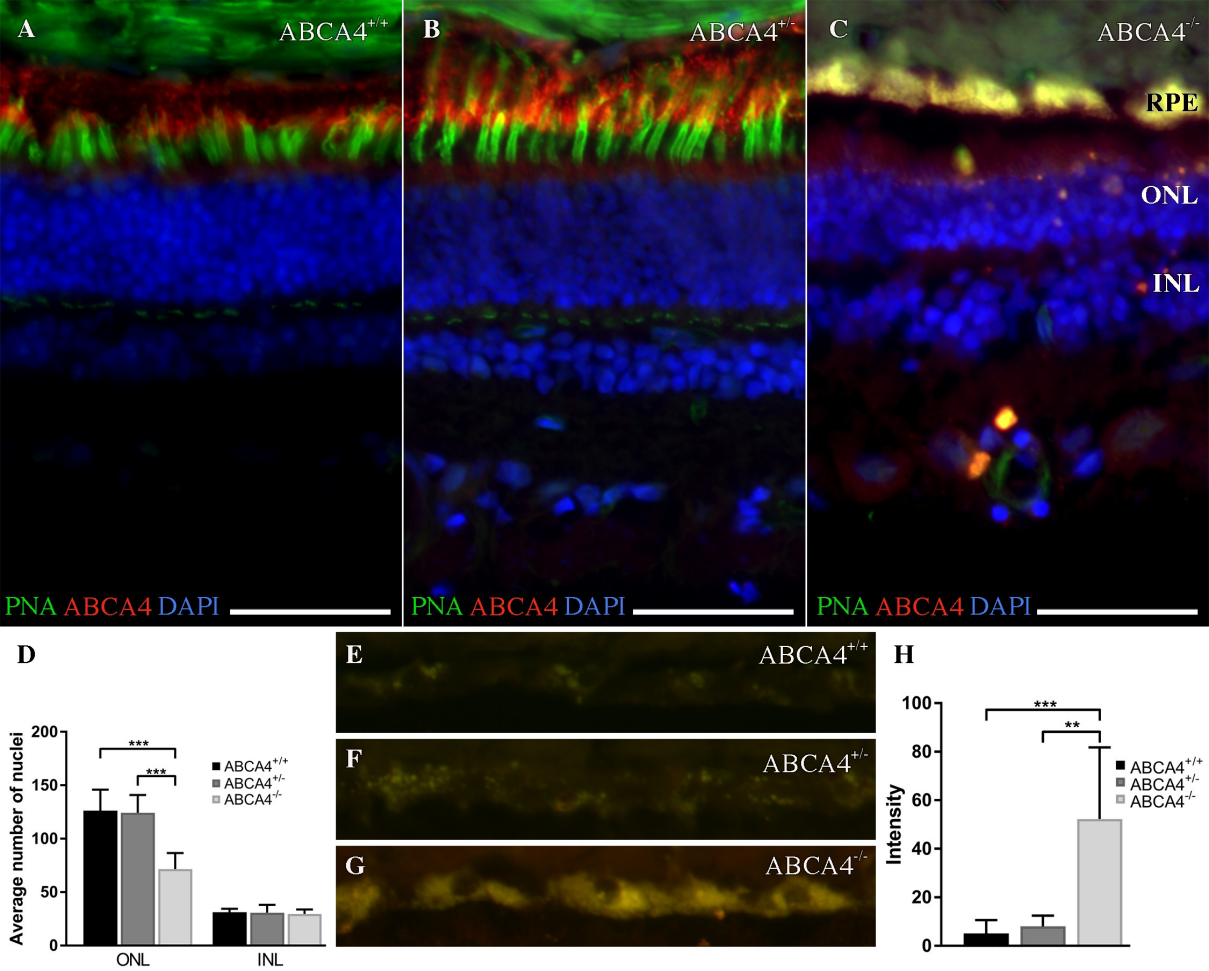
Fluorescence histochemistry of ABCA4, cone photoreceptors, and autofluorescence in the canine retina. **(A-C)** Fluorescence micrographs showing ABCA4 expression (red), FITC-conjugated peanut agglutinin (PNA, green), and DAPI nuclear staining (blue) in wild-type (*ABCA4*^*+/+*^), heterozygous (*ABCA4*^*+/-*^), and affected (*ABCA4*^*-/-*^) retinas. PNA labels cone photoreceptors. Autofluorescence, indicative of lipofuscin accumulation, was seen in the *ABCA4*^*-/-*^ RPE. **(D)** Bar graph with the average number of DAPI-stained nuclei within a given region of the ONL and the INL. **(E-G)** Fluorescence micrographs of RPE without immunohistochemistry show autofluorescence. **(H)** Bar graph with background-corrected mean autofluorescence-intensity in the RPE. Note the reduction of ABCA4-immunoreactivity and PNA binding, higher autofluorescence, and fewer nuclei in the ONL in the *ABCA4*^*-/-*^ compared to *ABCA4*^*+/+*^ or *ABCA4*^*+/-*^ retinas. All scale bars = 50 μm; RPE = retinal pigment epithelium; ONL = outer nuclear layer; INL = inner nuclear layer; Because there was only one individual per genotype, the statistics are valid for the technical replicates. ANOVA with Tukey’s post hoc test, n = 6; **P < 0.01; ***P < 0.001; mean ± S.D.

Fluorescence histochemistry was used to analyze the ABCA4 and rhodopsin protein expression in retinas from three dogs with different *ABCA4* genotypes. In addition, we used peanut agglutinin (PNA) as it selectively binds to cone photoreceptors [31]. Consistent with the western blot results, rhodopsin immunoreactivity (IR) was detected in the outer segments of rod photoreceptors in all three retinas (**S2 Fig**). In the wild-type (LAB26) and the heterozygous dog (LAB6), the ABCA4 IR was seen in the outer segments of the neural retina and in the RPE (**Fig 4A and 4B**). The ABCA4 IR was partially overlapping with the PNA staining, observed in both the inner and outer segments of the cone photoreceptor cells (**Fig 4A and 4B**). In sharp contrast, ABCA4 expression was absent and only a limited PNA staining was observed in the retina of the affected dog (LAB4; **Fig 4C**). The observed staining pattern in the fluorescence histochemistry thus suggested loss of cone photoreceptors.

To quantify photoreceptor degeneration in the retina of the affected dog (LAB4), we counted nuclei in the outer and inner nuclear layers and compared the results from the three genotypes. The photoreceptor nuclei are positioned in the outer nuclear layer (ONL) and the inner nuclear layer (INL) is composed of the horizontal, bipolar, amacrine and Müller glia cell nuclei. Approximately, a 46% reduction of the number of nuclei in the ONL was observed in the affected retina compared to the wild-type (LAB26) and heterozygous (LAB6) retinas (**Fig 4D**). Thus, the reduction of nuclei in the ONL supported a reduction of the number of photoreceptors. The results from the IR and PNA stainings had already shown a profound reduction of cone photoreceptors, but to assess whether rods were also degenerated in the affected retina, we inferred the number of rod photoreceptors in the wild-type and heterozygous retinas by substracting the number of cone nuclei from the total number of nuclei in the ONL. Approximately, a 41% reduction of rod nuclei was observed in the affected retina, consistent with a retinal degeneration involving also rod photoreceptors (**S2 Fig**). The corresponding reduction of nuclei was not seen in the INL, suggesting that photoreceptors were affected but not neurons in the INL. Taken together, we observed loss of ABCA4 protein, profound reduction of cone outer segment PNA staining, and a reduction of photoreceptor nuclei in the affected retina. The observed reduction in both cone and rod nuclei imply that not only cone photoreceptors but also rod photoreceptors degenerate in the *ABCA4*^-/-^ retina of these dogs.

The RPE layer of the affected retina was autofluorescent (**Fig 4C**), indicating accumulation of lipofuscin [32]. We estimated the intensity of autofluorescence in RPE from retinas representing the three *ABCA4* genotypes (LAB4, LAB6 and LAB26). The autofluorescence in the affected retina was approximately seven-fold higher compared to the retinas of the other genotypes (**Fig 4G and 4H**).

**Fig 5:**
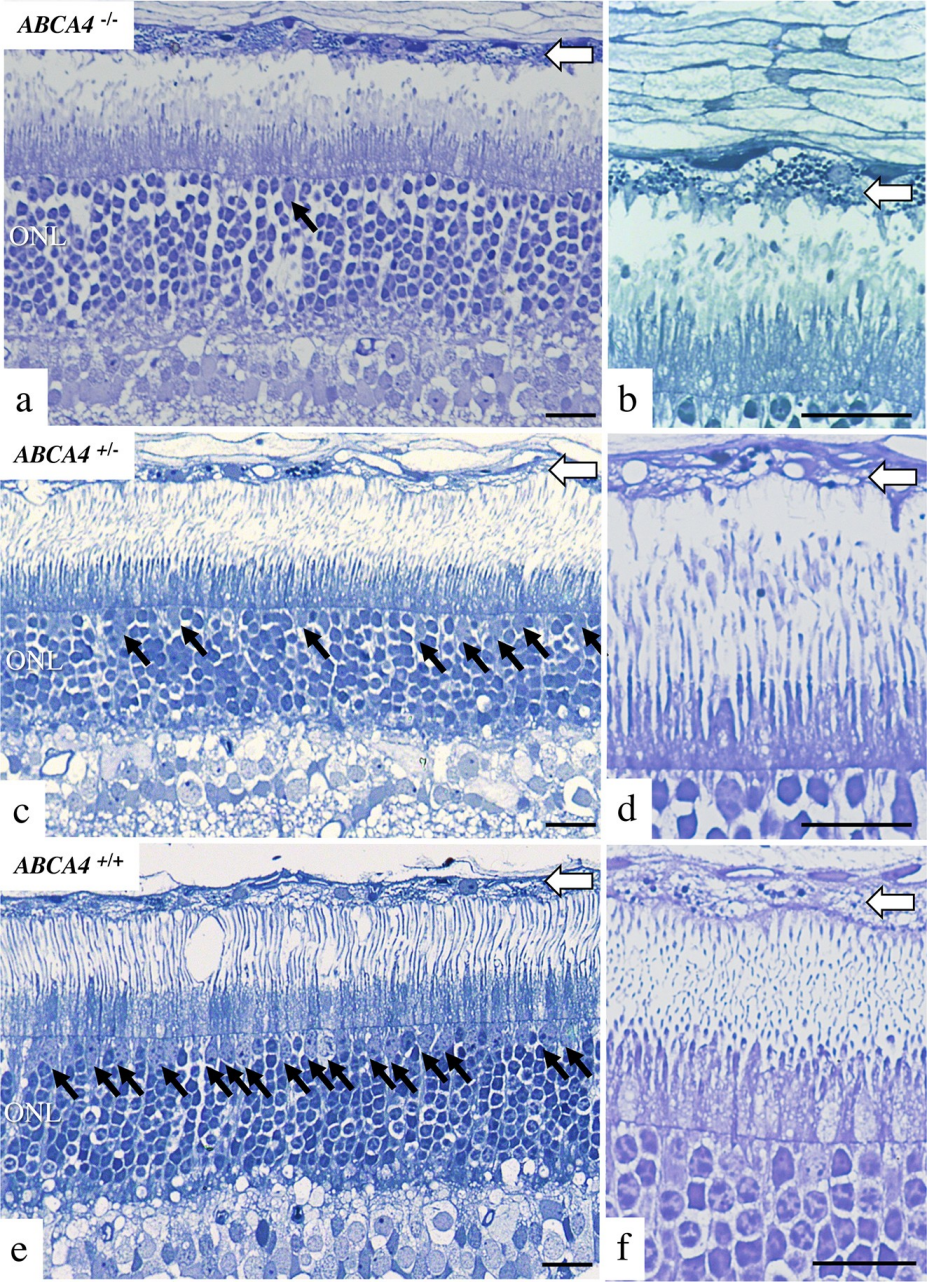
Histopathology. Light microscopic histology of a 12-year-old affected (LAB4) dog **(A, B)**, 12-year old heterozygote (LAB6) dog **(C, D)** and 10-year old unaffected (German spaniel) dog **(E, F)** taken at comparable locations in the superior central retina (0.5–1.5 cm dorsal to the optic nerve on the sagittal plane). Cone photoreceptors in the retina of the affected dog **(A)** were scarce (cone nuclei indicated with black arrow) compared with the retinas of the heterozygote **(C)** and wild-type **(E)** dogs. In the affected dog **(B)**, accumulation of lipofuscin was abundant in retinal epithelial cells (thick white arrow), compared to heterozygote **(D)** and wild-type **(F)** dogs. Photoreceptor outer segment disruption is artifactual. All scale bars = 20 microns; ONL = outer nuclear layer.

Light microscopic histopathology (**Fig 5**) was performed on retina from the affected dog (LAB4), a heterozygote (LAB6) and an unaffected dog (German spaniel). We examined plastic embedded thick sections taken from tapetal and non-tapetal regions superior and nasal to the optic nerve. An accumulation of round lipophilic bodies was found in the RPE overlying the tapetal region of the affected retina (**Fig 5B**). In contrast to the pigmented RPE in humans, dogs have a reflective area, the tapetum lucidum, in the choroid, where the overlying RPE is not pigmented [33]. The observed round lipophilic bodies predominantly seen in the affected dog are therefore not likely to be melanosomes, but rather an accumulation of lipofuscin. This is consistent with the increased intensity of autofluorescence observed in affected retina as described above (**Fig 4G and 4H**). In the nasal, non-tapetal part of the retina of the affected male, we observed multifocal RPE hyperplasia and hypertrophy, accompanied by overlying retinal atrophy in some, but not all of these foci (**S3 Fig**). Consistent with the reduction of cone photoreceptors observed in the frozen sections (**Fig 4D; S2 Fig**), cone nuclei were markedly reduced in the affected dog (**Fig 5A**) compared to heterozygote and control retinas. Reduced ONL thickness could not be unambiguously confirmed, however it should be noted that very short segments of retina were used for plastic embedding, and that regional ONL atrophy could therefore not be ruled out. In conclusion, histopathologic comparison identified increased lipofuscin accumulation in the RPE, cone loss in central superior retina and focal RPE hypertrophy and hyperplasia in nasal retina of the affected dog.

**Fig 6:**
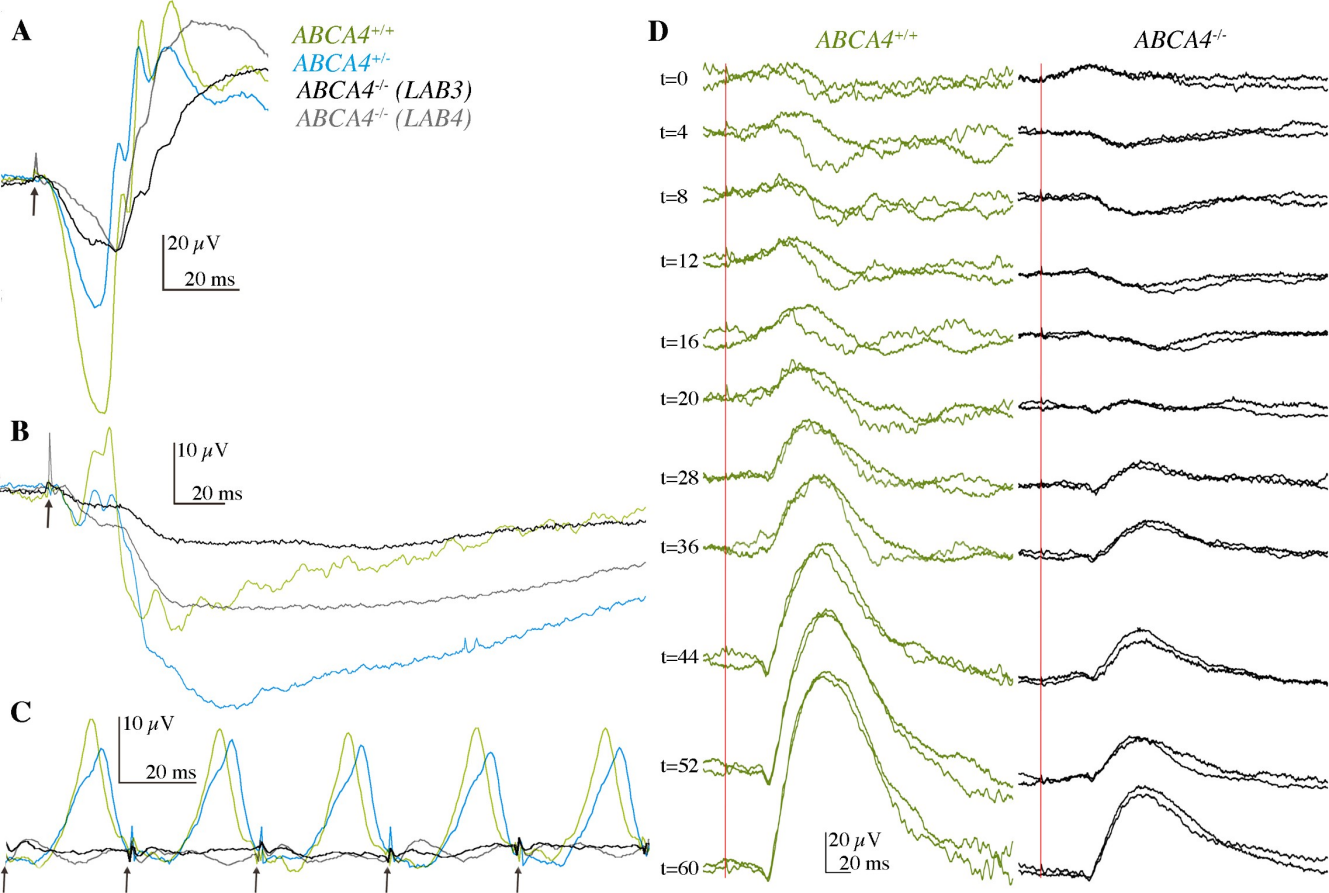
Flash-electroretinography (FERG) were used to assess retinal function *in vivo*. The green, blue, grey and black tracings indicate wild-type (LAB22; *ABCA4*^+/+^) unaffected dog, a heterozygous (LAB6; *ABCA4*^+/-^) dog, and two homozygous (LAB4 and LAB3; *ABCA4*^-/-^) affected dogs, respectively. Black arrows in A, B and C indicate 3 cd/m^2^/s-flash stimuli and the red line in D indicate 0.02 cd/m^2^/s-flash stimuli and scales show amplitude on the y-axis (μV) and time in ms on the x-axis for each type of response. **(A)** A dark-adapted, mixed rod-cone response. **(B)** Light-adapted cone transient responses **(C)** and cone flicker response at 30 Hz. Note that the affected dog had a delayed response to the stimuli. **(D)** The dark-adapted rod responses monitored during one hour in an affected (LAB3) and a wild-type female (LAB22).

We used flash-electroretinography (FERG) to study the photoreceptor function in four dogs at the age of 10 years. The inclination of the first part of the a-waves of the dark-adapted FERG in response to a bright stimulus was less steep and the amplitudes of the a-waves were lower in both affected dogs (LAB3 and LAB4) and their heterozygous sibling (LAB6), as compared to the age-matched, unaffected dog (LAB22) (**Fig 6A**), suggesting abnormal photoreceptor function in the affected dogs. The light-adapted FERG responses were subnormal for the affected dogs, showing profoundly impaired cone function (**Fig 6B and 6C**). The light-adapted responses of the heterozygous dog were closer to the wild-type dog, although amplitudes were slightly lower and b-wave and flicker implicit times slightly longer (**Fig 6B and 6C**). Furthermore, dark-adaptation reflecting rod photoreceptor function, was clearly delayed in the affected dog (**Fig 6D**). After 20 minutes, the time commonly used for dark-adaptation [34], the rod responses of the affected dogs had very low amplitudes. After one hour of dark-adaptation, the affected male (LAB4) reached near normal amplitudes, whereas the amplitudes of his female sibling (LAB3) remained clearly subnormal (**Fig 6D**), showing that the rod photoreceptors were also affected, but their function was better preserved than the function of the cone photoreceptors.

**Fig 7:**
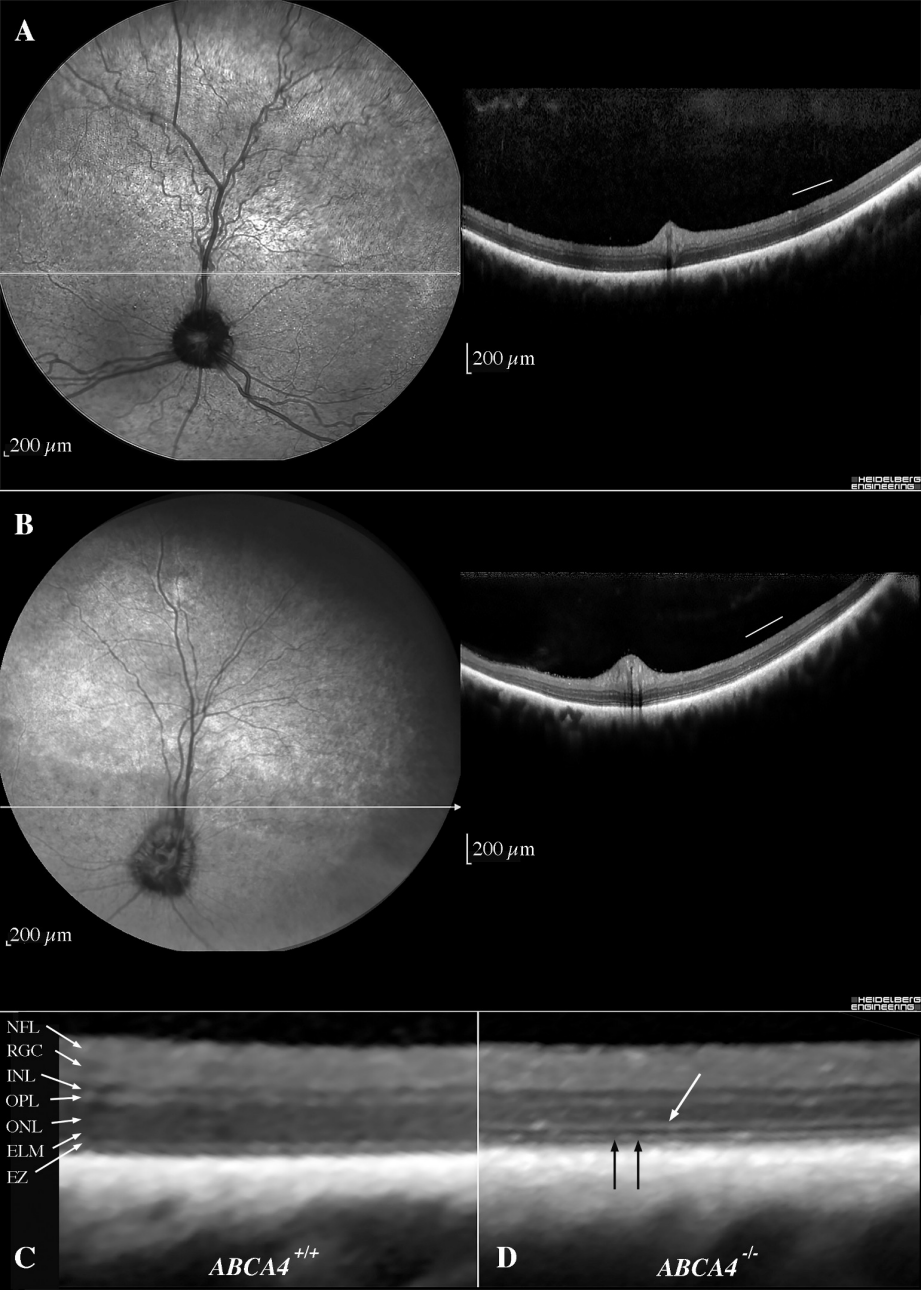
*In vivo* retinal morphology assessed with cSLO and OCT. cSLO-images (left) and OCTs (right) **(A)** from the left eye of a 12-year-old unaffected dog (LAB22) along the visual streak and **(B)** from the left eye of a 10-year-old affected dog (LAB10), where the horizontal extension of the visual streak is indicated by the black arrows. **(C)** A magnification of the temporal retina (corresponding to the area below the white bar in the OCTs) of the unaffected dog (LAB22) and accordingly **(D)** of the affected dog (LAB10), with thickened and hyperreflective ELM (white arrow) and fragmented EZ (black arrows). cSLO = confocal scanning laser ophthalmoscopy; OCT = optical coherence tomography; ELM = external limiting membrane; EZ = ellipsoid zone (inner-to-outer segment junction).

**Fig 8:**
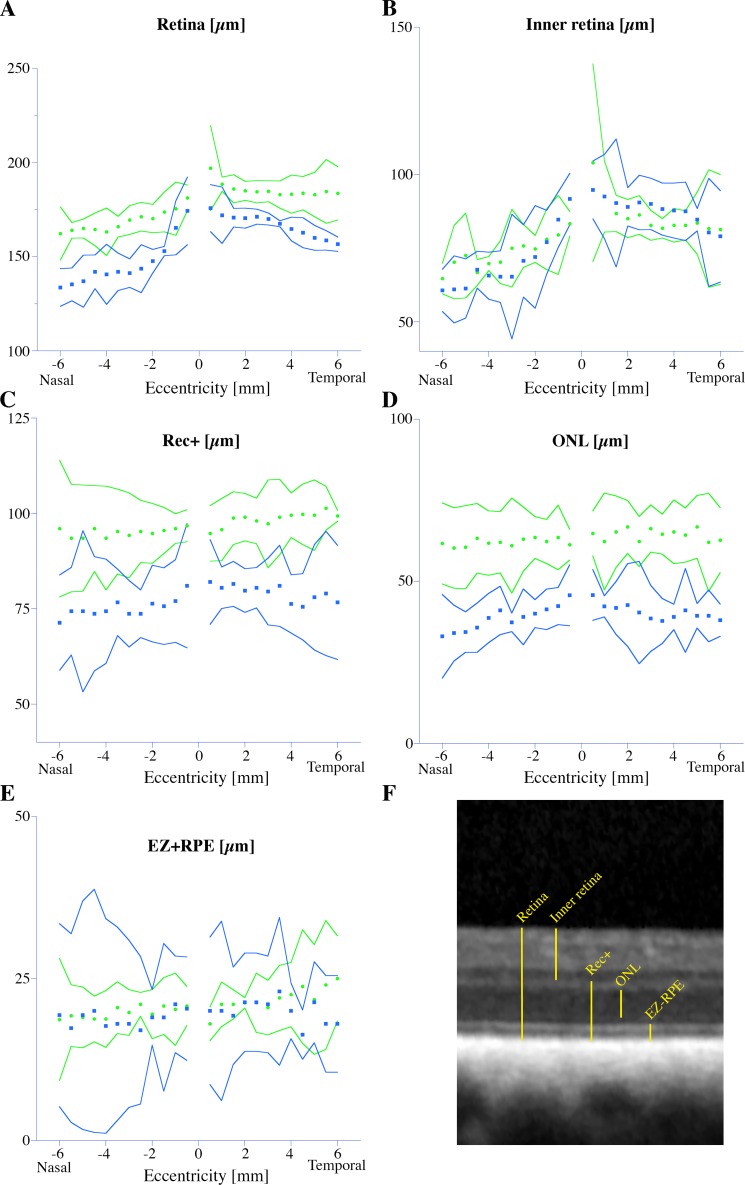
Analysis of retinal layer thickness using OCT. Graphs showing the mean thickness of retinal layers from OCTs of two wild-type dogs at the age of 10- and 12-years (green dots; LAB23 and LAB22, respectively) and two affected dogs at the age of 10- and 12-years (blue squares; LAB10 and LAB16, respectively). The thickness is measured every 0.5 mm from the rim of the optic nerve head (0) along the visual streak. The solid lines indicate 95% confidence intervals. Measurement of the **(A)** total retinal thickness, **(B)** inner retina **(C)** Rec+, **(D)** ONL and **(E)** EZ+RPE. **(F)** The distances presented in the five graphs are shown on the magnified OCT image from a wild-type Labrador retriever dog. OCT = optical coherence tomography; EZ = ellipsoid zone (inner-to-outer segment junction); Rec+ = total photoreceptor length; RPE = retinal pigment epithelium; ONL = outer nuclear layer.

Optical coherence tomography (OCT) was performed along the visual streak in three Labrador retriever dogs (**S4 Fig**). The affected dog (LAB4) had a thinner retina with marked reduction in ONL thickness. Furthermore, we observed some areas of full-thickness retinal atrophy, where the retinal layers could not be distinguished. We were unable to link the areas of alternating normal to grayish hyporeflectivity observed ophthalmoscopically (**Fig 1**) to localized retinal lesions on OCT. The abnormal and variable tapetal reflectivity seen on ophthalmoscopy was therefore considered to be a sign of a diffusely spread degeneration altering the translucency of the retina overlying the tapetum lucidum. Additional examinations using confocal scanning laser ophthalmoscopy (cSLO) and OCT imaging of two affected dogs at the age of 10- and 12-years (LAB10 and LAB16, respectively) confirmed a thinning of the outer retina along the visual streak as compared to two age-matched wild-type dogs (LAB22 and LAB23) (**Figs 7 and 8**). Compared to the wild-type dog (LAB22) (**Fig 7A**), a more irregular tapetal reflection with a hyporeflective visual streak and vascular attenuation was observed on the cSLO of the affected dog (LAB10) (**Fig 7B**). The thickness of the INL was similar in both the wild-type and the affected dogs (**Fig 7C and 7D**). The external limiting membrane was thickened and hyperreflective (**Fig 7D**), whereas the ellipsoid zone (EZ), which corresponds to the junction between the outer and inner segments of the photoreceptors, was fragmented (**Fig 7D**). The total retinal thickness (**Fig 8A**) was markedly reduced in both affected Labrador retriever dogs (LAB10 and LAB16) compared to the wild-type dogs (LAB22 and LAB23). However, measurements of the inner retina (**Fig 8B**) showed similar thickness in this part of the retina in all four dogs analyzed. Total photoreceptor length (REC+; **Fig 8C**) and the thickness of the ONL (**Fig 8D**) were markedly reduced both nasally and temporally in the affected dogs, showing that the degeneration of the outer retina is not confined only to the area centralis. The average distance from the EZ to the RPE/Bruch’s membrane (the innermost layer of the choroid) was similar in both genotypes (**Fig 8E**).

Taken together, vision of the affected dogs at the age of 10 to 12 years was impaired in both daylight and dimlight conditions, but they still retained some vision throughout their lifetime. The clinical features included ophthalmoscopic signs of bilateral diffuse retinal degeneration and *in vivo* morphology indicaded a reduction of the number of photoreceptors. The cone function was profoundly abnormal, whereas rod function was better preserved. A hallmark of human *ABCA4*-mediated diseases such as STGD, is the accumulation of autofluorescent lipofuscin in the RPE throughout the fundus [32, 35]. This is also seen in mouse models [36, 37] as well as in the canine retinal degenerative disease described here. In addition, cone photoreceptors are typically affected prior to rods [38]. Furthermore, human RPE cells have been shown to be hypertrophic, and at more advanced stages of the disease, RPE is lost in the perifovea [39, 40]. Similar to the human histopathology, we observed accumulation of autofluorescent lipofuscin, regions of RPE hypertrophy and hyperplasia, as well as thinning of ONL in the affected dog.

Mutations in the human *ABCA4* (*ABCR*) gene cause several clinically different diseases ranging from autosomal recessive STGD and autosomal recessive forms of CRD to RP [41–43]. The severity of the disease phenotype is suggested to be dependent on the severity of the mutations [41]. The gene was first cloned and characterized in 1997 [21], and to date, 873 missense and 58 loss-of-function variants have been reported in the ExAC database [44, 45], many of which are associated with visual impairment [46–48].

The ABCA4 protein functions as an ATP-dependent flippase in the visual cycle, transporting *N*-retinylidene-phosphatidylethanolamine (*N*-Ret-PE) from the photoreceptor disc lumen to the cytoplasmic side of the disc membrane [49, 50]. *N*-Ret-PE is a reversible adduct spontaneously formed between all-*trans*-retinal and phosphatidylethanolamine (PE), and is unable to diffuse across the membrane by itself. Once transported by ABCA4, *N*-Ret-PE is dissociated and all-*trans*-retinal will re-enter the visual cycle [51]. Defective ABCA4 leads to accumulation of *N*-Ret-PE, which together with all-*trans*-retinal, will form di-retinoid-pyridinium-phosphatidylethanolamine (A2PE) that is further hydrolyzed to phosphatidic acid (PA) and a toxic bis-retinoid, di-retinal-pyridinium-ethanolamine (A2E) [52]. This will lead to an accumulation of A2E in RPE cells when photoreceptor discs are circadially shed and phagocytosed by the RPE [36, 53, 54]. A2E is a major component of RPE lipofuscin, accounts for a substantial portion of its autofluorescence, and has a potentially toxic effect on the RPE leading to photoreceptor degeneration [36, 55–57].

Currently, there is no standard treatment for STGD in humans and mouse is the only available animal model [58, 59]. Both the *Abca4* knockout mouse [36] and the recently generated *Abca4* p.Asn965Ser (N965S) knockin mouse [37] models have been significant for the functional characterization of ABCA4 and the lipofuscin fluorophore A2E. Mice, however, lack the macula, the area primarily affected in STGD patients and no significant retinal degeneration has been observed in any of the mouse models [37, 60, 61]. Unlike the mouse retina, the dog has a cone rich, fovea-like area functionally more similar to human fovea centralis [2, 10, 11]. The canine eye is also comparable in size to the human eye, and dog models have successfully been used for experimental gene therapy for retinal degenerative diseases, such as LCA, RP, and rod-cone dysplasia type 1 (rcd1) [12, 14, 16, 62]. For over a decade there has been interest in finding a canine model for *ABCA4*-mediated diseases [23, 63, 64]. The loss-of-function mutation identified here can be used to develop a large animal model for human STGD.

## Materials and methods

### Animals and samples

A family quartet of Labrador retriever dogs (sire, dam, and two affected offspring numbered LAB1, LAB2, LAB3, and LAB4, respectively) were used in the whole-genome sequencing (WGS). In addition, 16 related individuals (LAB5 to LAB20, see **S1 Fig**) as well as six unrelated Labrador retrievers (LAB 21 to LAB26) were used to validate the WGS findings. Whole blood samples from these dogs were collected in EDTA tubes and genomic DNA was extracted using 1 ml blood on a QIAsymphony SP instrument and the QIAsymphony DSP DNA Kit (Qiagen, Hilden, Germany). We obtained eyes from the affected male (LAB4) and his unaffected sibling (LAB6) at the age of 12, as well as from two unrelated, unaffected female Labrador retrievers (LAB24 and LAB26, 11- and 10-year-old, respectively) and one 10-year-old male German spaniel (GS) after euthanasia with sodium pentobarbithal (Pentobarbithal 100 mg/ml, Apoteket Produktion & Laboratorier AB, Stockholm, Sweden) for reasons unrelated to this study. All samples were obtained with informed dog owner consent. Ethical approval was granted by the regional animal ethics committee (Uppsala djursförsöksetiska nämnd; Dnr C12/15 and C148/13).

### Ophthalmic examination

Ophthalmic examination of all the dogs included in the study included reflex testing, testing of vision with falling cotton balls under dim and daylight conditions, as well as indirect ophthalmoscopy (Heine 500, Heine Optotechnik GmbH, Herrsching, Germany) and slit-lamp biomicroscopy (Kowa SL-15, Kowa Company Ltd., Tokyo, Japan) after dilation of pupils with tropicamide (Mydriacyl 0.5%, Novartis Sverige AB, Täby, Sweden).

### Whole-genome sequencing

Genomic DNA from four Labrador retriever dogs (LAB1, LAB2, LAB3 and LAB4) was fragmented using the Covaris M220 instrument (Covaris Inc., Woburn, MA), according to the manufacturer’s instructions. To obtain sufficient sequence depth, we constructed two biological replicates of libraries with insert sizes of 350 bp and 550 bp following TruSeq DNA PCR-Free Library Prep protocol. The libraries were multiplexed and sequenced on a NextSeq500 instrument (Illumina, San Diego, CA) for 100 x 2 and 150 x 2 cycles using the High Output Kit and High Output Kit v2, respectively. The raw base calls were de-multiplexed and converted to fastq files using bcl2fastq v.2.15.0 (Illumina). The two sequencing runs from each individual were merged, trimmed for adapters and low-quality bases using Trimmomatic v.0.32 [65], and aligned to the canine reference genome CanFam3.1 using Burrows-Wheeler Aligner (BWA) v.0.7.8 [66]. Aligned reads were sorted and indexed using Samtools v.1.3 [67] and duplicates were marked using Picard v.2.0.1. The BAM files were realigned and recalibrated with GATK v.3.7 [68]. Multi-sample variant calling was done following GATK Best Practices [69] using publicly available genetic variation Ensembl Variation Release 88 in dogs (*Canis lupus familiaris*). We filtered the variants found by GATK using the default values defining two groups of analyses: trio 1 and 2, both consisting of the same sire and dam, and one of their affected offspring. Variants annotated in the exonic region with ANNOVAR v.2017.07.16 [70], presenting an autosomal recessive inheritance pattern and shared between the two trios were selected for further evaluation. To predict the effects of amino acid changes on protein function, we evaluated SNVs using PolyPhen-2 v2.2.2r398 [71] and PROVEAN v.1.1.3 [72] and non-frameshift INDELS using PROVEAN v.1.1.3. Frameshift INDELs were manually inspected using The Integrative Genomics Viewer (IGV) [73, 74]. The sequence data were submitted to the European Nucleotide Archive with the accession number PRJEB26319.

### Validation of the variants

To validate the WGS results, we designed primers amplifying the variants c.7244C>T in *USH2A* gene and c.4176insC in *ABCA4* gene with Primer3 [75, 76] (**S5 Table**) and sequenced the family quartet using Applied Biosystems 3500 Series Genetic Analyzer (Applied Biosystems, Thermo Fisher Scientific, Waltham, MA). To test if the variants were concordant with the disease, 22 additional ophthalmologically evaluated Labrador retrievers were genotyped by Sanger sequencing (**S1 Fig**). Eight of these dogs were clinically affected and fourteen were unaffected, showing no signs of retinal degeneration by seven years of age.

### Quantitative RT-PCR (qPCR)

Neuroretinal samples were collected from the affected dog (LAB4), the heterozygous sibling (LAB6), and the unaffected female (LAB24). The samples were immediately preserved in RNAlater (SigmaAldrich, Saint Louis, MO), homogenized with Precellys homogenizer (Bertin Instruments, Montigny-le-Bretonneux, France) and total RNA was extracted with RNAeasy mini kit (Qiagen) according to the manufacturer’s instructions. RNA integrity and quality were inspected with Agilent 6000 RNA Nano kit with the Agilent 2100 Bioanalyzer system (Agilent Technologies, Santa Clara, CA). cDNA was synthesized using RT^2^ First Strand kit (Qiagen) with random hexamers provided in the kit. cDNA concentration was inspected with Qubit ssDNA Assay kit (Life Technologies, Thermo Fisher Scientific). RT^2^ qPCR Primer Assay (Qiagen) was used to amplify the reference gene *GAPDH*. To amplify the target gene *ABCA4*, we designed custom primers with Primer3 [75, 76] targeting three different regions spanning exons 2 to 3, 27 to 28, and 47 to 48 (**S5 Table**). We amplified the cDNA fragments encoding regions of interest using RT^2^ SYBR Green ROX qPCR Mastermix (Qiagen) with StepOnePlus Real-Time PCR system (Applied Biosystems, Thermo Fisher Scientific), according to the manufacturer’s instructions. Target gene expression was normalized to expression of *GAPDH*, and shown relative to the unaffected female (LAB24) using the △△C_T_ method. The results were confirmed in two independent experiments.

### SDS-Gel Electrophoresis and Western Blotting

We extracted protein from the neuroretinal samples of the individuals used in qPCR (see above) by homogenization in Pierce RIPA lysis buffer (Thermo Scientific) supplemented with phosphatase inhibitor cocktail (Sigma, P8340) using the Precellys homogenizer (Bertin Instruments). Protein concentration was determined using the Pierce BSA Protein Assay kit (Thermo Fisher Scientific). 50 μg of protein samples were resolved by SDS-PAGE, transferred to nitrocellulose membrane, and immunoblotted with the following primary antibodies: ABCA4 (Novus Biologicals, NBP1-30032, 1:1000), GAPDH (Thermo Scientific, MA5-15738, 1:1000), Rhodopsin (Novus Biologicals, Littleton, CO, NBP2-25160H, 1:5000), followed by Anti-Mouse IgG horseradish peroxidase-conjugated secondary antibody (R&D Systems, HAF007, 1:5000). Binding was detected using the Clarity western ECL substrate (Bio-Rad, Hercules, CA).

### Fluorescence histochemistry

Tapetal fundus from the affected male (LAB4), his unaffected heterozygous sibling (LAB6), and an unaffected 10-year-old female Labrador retriever (LAB26) were fixed in 4% PFA in 1x PBS on ice for 15 minutes, washed in 1x PBS for 10 minutes on ice, and cryoprotected in 30% sucrose overnight at 4°C. The central part of the fundus was embedded in Neg-50™ frozen section medium (Thermo Scientific), and 10 μm sections from the tapetal part of the eye were collected on Superfrost Plus slides (J1800AMNZ, Menzel-Gläser, Thermo Fisher Scientific). The sections were re-hydrated in 1x PBS for 10 minutes, incubated in blocking solution (1% donkey serum, 0.02% thimerosal, and 0.1% Triton X-100 in 1x PBS) for 30 minutes at room temperature, and incubated in primary antibody ABCA4 (1:500, NBP1-30032, Novus Biologicals) or rhodopsin (1:5000, NBP2-25160, Novus Biologicals), and FITC-conjugated lectin PNA (1:400, L21409, Molecular Probes) solution at 4°C overnight. Following overnight incubation, the slides were washed 3 x 5 minutes in 1x PBS and incubated in Alexa 568 secondary antibody (1:2000, A10037, Invitrogen, Thermo Fisher Scientific) solution for at least 2 hours at room temperature and washed 3 x 5 minutes in 1x PBS. The slides were mounted using ProLong Gold Antifade Mountant with DAPI (P36931, Molecular Probes, Thermo Fisher Scientific). Fluorescence images were captured using a Zeiss Axioplan 2 microscope equipped with an AxioCam HRc camera.

### Counting nuclei

Ten micrometer retinal sections were stained and mounted as described under Fluorescence histochemistry, and the number of nuclei within a region with a width of 67 μm that was perpendicular to and covered both the outer and inner nuclear layers were counted. Nuclei in the outer nuclear and inner nuclear layers were counted separately. We inferred the number of rod photoreceptors by subtracting the number of cones, as identified by PNA staining, from the number of nuclei in the ONL. We analyzed six images from each of the three dogs (LAB4, LAB6, and LAB26). Note that cones were so rare in the affected retina, that all the nuclei in the ONL represent rod photoreceptors. Bar graphs were generated and statistical analysis of the technical replicates (one-way ANOVA with Tukey’s post hoc multiple comparison analysis) was performed in GraphPad Prism 7.

### Autofluorescence

Retinal sections were washed, incubated in blocking solution, and mounted as described under Fluorescence histochemistry. The exposure times for the excitation at 488 nm and 568 nm were fixed for all images taken (150 ms and 80 ms, respectively). Outlines of the retinal pigment epithelium (RPE), as well as adjacent background regions, were drawn using the polygon selection tool in ImageJ (v1.51, NIH), and the area and mean fluorescence intensity were measured. The mean intensity of the autofluorescence in the RPE was calculated by subtracting the background intensity from the adjacent regions. We analyzed six images from each of the three individuals used in the fluorescence histochemistry. Bar graph generation and statistical analysis were performed as described under Counting nuclei.

### Histopathology

Light microscopic examination was performed on plastic embedded thick sections from 4% PFA fixed posterior sections from eyes of the affected male (LAB4) and his heterozygous sibling (LAB6), as well as from an unaffected 10-year-old German spaniel dog. The samples were post-fixed in 2.5% glutaraldehyde-2% formaldehyde (2 hours), 2% glutaraldehyde-1% osmium tetroxide (1.5 hours), and 2% osmium tetroxide (1.5 hours). The posterior segments were then trimmed into segments 2–5 mm in length, taken from the superior retina (three sections located 0.5 cm to 1.5 cm dorsal to the optic nerve), and the nasal retina (two sections from non-tapetal retina). These were dehydrated, and embedded in epoxy resin (PolyBed 812; Polysciences, Warrington, PA). Tissues were sectioned at 1μm and stained with azure II-methylene blue/paraphenylenediamine counterstain. Sections were examined with a 40× objective on a light microscope (Axioplan; Carl Zeiss Meditec GmbH Oberkochen, Germany) and images collected with an AxioCam MrC digital camera (Carl Zeiss Meditec GmbH Oberkochen, Germany).

### Flash-electroretinography (FERG)

We recorded full-field FERG from the four dogs (LAB3, LAB4, LAB6 and LAB22) examined with OCT under general anaesthesia. Sedation with intramuscular acepromazine 0.03 mg/kg (Plegicil vet., Pharmaxim Sweden AB) was followed by induction with propofol 10 mg/kg intravenously (Propovet, Orion Pharma Animal Health AB, Danderyd, Sweden). After intubation, inhalation anaesthesia was maintained with isoflurane (Isoflo vet., Orion Pharma Animal Health AB). Corneal electrodes (ERG-JET, Cephalon A/S, Aalborg, Denmark) were used with isotonic eye drops (Comfort Shield, i.com medical GmbH, Munich, Germany) as coupling agent. Gold-plated, cutaneous electrodes served as ground and reference electrodes (Grass, Natus Neurology Inc. Pleasanton, CA) at the vertex and approximately 3 cm caudal to the lateral canthi, respectively. Light stimulation, calibration of lights, and processing of signals were performed as described by Karlstam et al., 2011 [77]. We used a slightly modified ECVO protocol [34], where the process of dark-adaptation was monitored for 1 hour before a dark-adapted response intensity series was performed.

### Optical coherence tomography (OCT)

The affected male (LAB4), his unaffected sibling (LAB6) and an unaffected, age-matched, female Labrador retriever (LAB22) were examined with spectral-domain OCT (Topcon 3D OCT-2000, Topcon Corp., Tokyo, Japan). The examination was done after pupillary dilation, but without sedation, using repeated horizontal single line scans (6 mm, 1024 A-scans) along the visual streak area. Additional cSLO- and OCT-imaging was performed in two affected (LAB10 and LAB16) and two unaffected, wild-type dogs (LAB22 and LAB23) using a Spectralis HRT + OCT Heidelberg Engineering GmbH, Germany). The dogs were lightly sedated with 0.01 mg medetomidine per kg intramuscularly (Sedator vet., Dechra Veterinary Products AB, Upplands-Väsby, Sweden), and corneas were kept moist using artificial tears (Aptus SentrX, Orion Pharma Animal Health, Danderyd, Sweden).

## Supporting information

S1 Fig Pedigree of the Labrador retriever dogs used in the study. Filled symbols indicate affected individuals, half-filled symbols represent obligate or genotyped carriers of the *ABCA4* insertion. Individuals LAB1 to LAB4 were used in the WGS analysis. Numbered individuals were genotyped for the insertion in the *ABCA4* gene (c.4176insC) and for the non-synonymous substitution in the *USH2A* gene (c.7244C>T). Crosses intersecting the dashed lines indicate the number of generations between the individuals.
(PPTX)

S2 Fig Rhodopsin expression and the prevalence of rod photoreceptors in the canine retina. **(A)** Fluorescence micrographs showing rhodopsin expression (red) in the *ABCA4*^*+/+*^ (left), *ABCA4*^*+/-*^ (middle), and *ABCA4*^*-/-*^ (right) rod outer segments. Scale bar = 10 μm. **(B)** Inferred number of rod photoreceptors based on the number of nuclei in the outer nuclear layer and the number of cone photoreceptors within a given region of the retina. Cone photoreceptors were identified via PNA, which binds selectively to cone photoreceptors. Because there was only one individual per genotype, the statistics are valid for the technical replicates. ANOVA with Tukey’s post hoc test, n = 6; ***P < 0.001; ****P < 0.0001; mean ± S.D.
(TIF)

S3 Fig Histologic changes in RPE in the affected individual. **(A-B)** Several focal regions of RPE hypertrophy (white arrows, **A**) as well as hyperplasia (black arrows, **A**, **B**), noted in two regions in the affected retina. Atrophy of overlying ONL and INL was noted over some (**A**; asterisk) of these regions. Lesions were focal (approximately 50–100 microns in diameter), intermittent and seen only in a section from nasal, nontapetal retina of the ABCA4^-/-^ dog. All scale bars = 100 microns.
(PDF)

S4 Fig OCT images along the visual streak. OCT scans from a 10-year old unaffected, wild-type dog (LAB22; top), a 12-year old heterozygous dog (LAB6; middle), and his affected littermate (LAB4; bottom). White arrows indicate where two images have been concatenated. A general thinning of ONL along the visual streak is visible in the affected retina compared to the wild-type and heterozygous retinas and included foci of severe retinal atrophy (red arrow).
OCT = optical coherence tomography; ONL = outer nuclear layer; ELM = external limiting membrane; EZ = ellipsoid zone (inner-to-outer segment junction); IZ = outer segment-RPE interdigitation zone.
(TIF)

S1 Table Summary of the whole-genome sequencing runs 1 and 2. (XLSX)

S2 Table Exonic variants identified in WGS. Number of exonic variants following autosomal recessive inheritance pattern (AR) in Trio1 and Trio2, each consisting of the parents and one of the two offspring. The total number of exonic variants in the family quartet including all inheritance patterns and the number of AR variants shared between the two trios. The "unique" column represents the number of AR variants, which were shared between the two trios and not found to be homozygous in 23 additional investigated canine genome sequences.
(XLSX)

S3 Table List of candidate variants from WGS. Coding sequence variants identified as private for the Labrador retriever family and the predicted effect of the variants based on Polyphen-2 and PROVEAN scores.
(XLSX)

S4 Table Validation of variants c.4176insC in ABCA4 gene and c.C7244T in USH2A gene by Sanger sequencing. (XLSX)

S5 Table Canine primer sequences used in the analysis. (XLSX)
