# Supplementary material for: An ABCA4 loss-of-function mutation causes a canine form of Stargardt disease
Source: PLoS Genet. 2019 Mar 19;15(3):e1007873. doi: 10.1371/journal.pgen.1007873 (PMC6424408; doi:10.1371/journal.pgen.1007873)
Supplement: S1 Fig — Filled symbols indicate affected individuals, half-filled symbols represent obligate or genotyped carriers of the ABCA4 insertion. Individuals LAB1 to LAB4 were used in the WGS analysis. Numbered individuals were genotyped for the insertion in the ABCA4 gene (c.4176insC) and for the non-synonymous substitution in the USH2A gene (c.7244C>T). Crosses intersecting the dashed lines indicate the number of generations between the individuals. (PPTX) [file pgen.1007873.s001.pptx]

## Slide 1
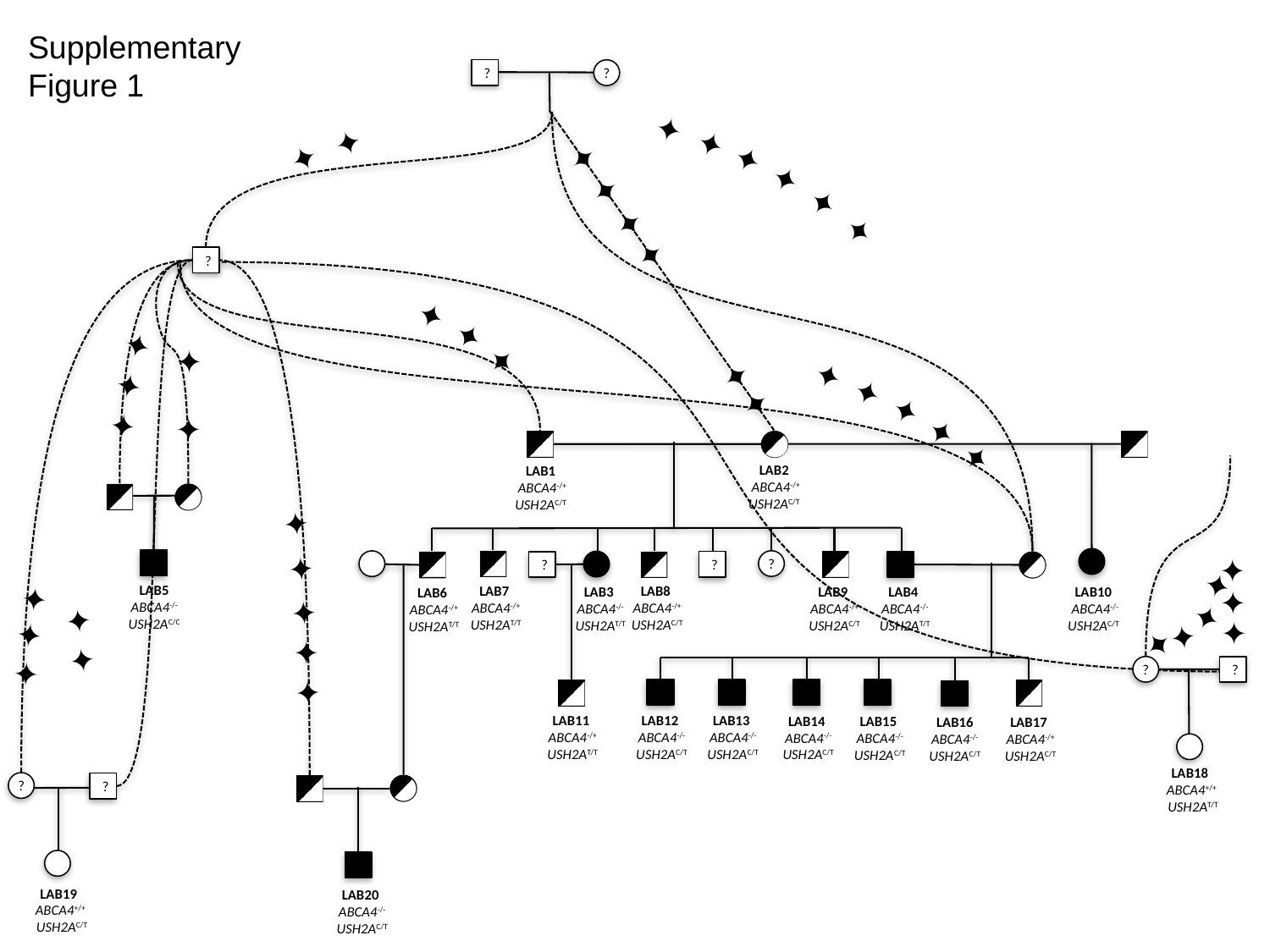

Supplementary
Figure 1
?
?
✦
✦
✦
✦
✦
✦
✦
✦
✦
✦
✦
✦
?
✦
✦
✦
✦
✦
✦
✦
✦
✦
✦
✦
✦
✦
✦
✦
LAB2
 ABCA4-/+
USH2AC/T
LAB1
 ABCA4-/+
USH2AC/T
✦
✦
✦
?
?
?
✦
✦
LAB5
ABCA4-/-
USH2AC/C
LAB8
 ABCA4-/+
 USH2AC/T
LAB7
 ABCA4-/+
 USH2AT/T
✦
LAB3
 ABCA4-/-
 USH2AT/T
LAB9
 ABCA4-/+
 USH2AC/T
LAB4
 ABCA4-/-
 USH2AT/T
LAB10
 ABCA4-/-
USH2AC/T
LAB6
 ABCA4-/+
 USH2AT/T
✦
✦
✦
✦
✦
✦
✦
✦
✦
✦
?
?
✦
LAB11
 ABCA4-/+
 USH2AT/T
LAB13
 ABCA4-/-
 USH2AC/T
LAB12
 ABCA4-/-
 USH2AC/T
LAB14
 ABCA4-/-
 USH2AC/T
LAB15
 ABCA4-/-
 USH2AC/T
LAB16
ABCA4-/-
 USH2AC/T
LAB17
 ABCA4-/+
 USH2AC/T
LAB18
 ABCA4+/+
 USH2AT/T
?
?
LAB19
 ABCA4+/+
 USH2AC/T
LAB20
 ABCA4-/-
 USH2AC/T
