# Supplementary figures and images for: An ABCA4 loss-of-function mutation causes a canine form of Stargardt disease
Source: PLoS Genet. 2019 Mar 19;15(3):e1007873. doi: 10.1371/journal.pgen.1007873 (PMC6424408; doi:10.1371/journal.pgen.1007873)

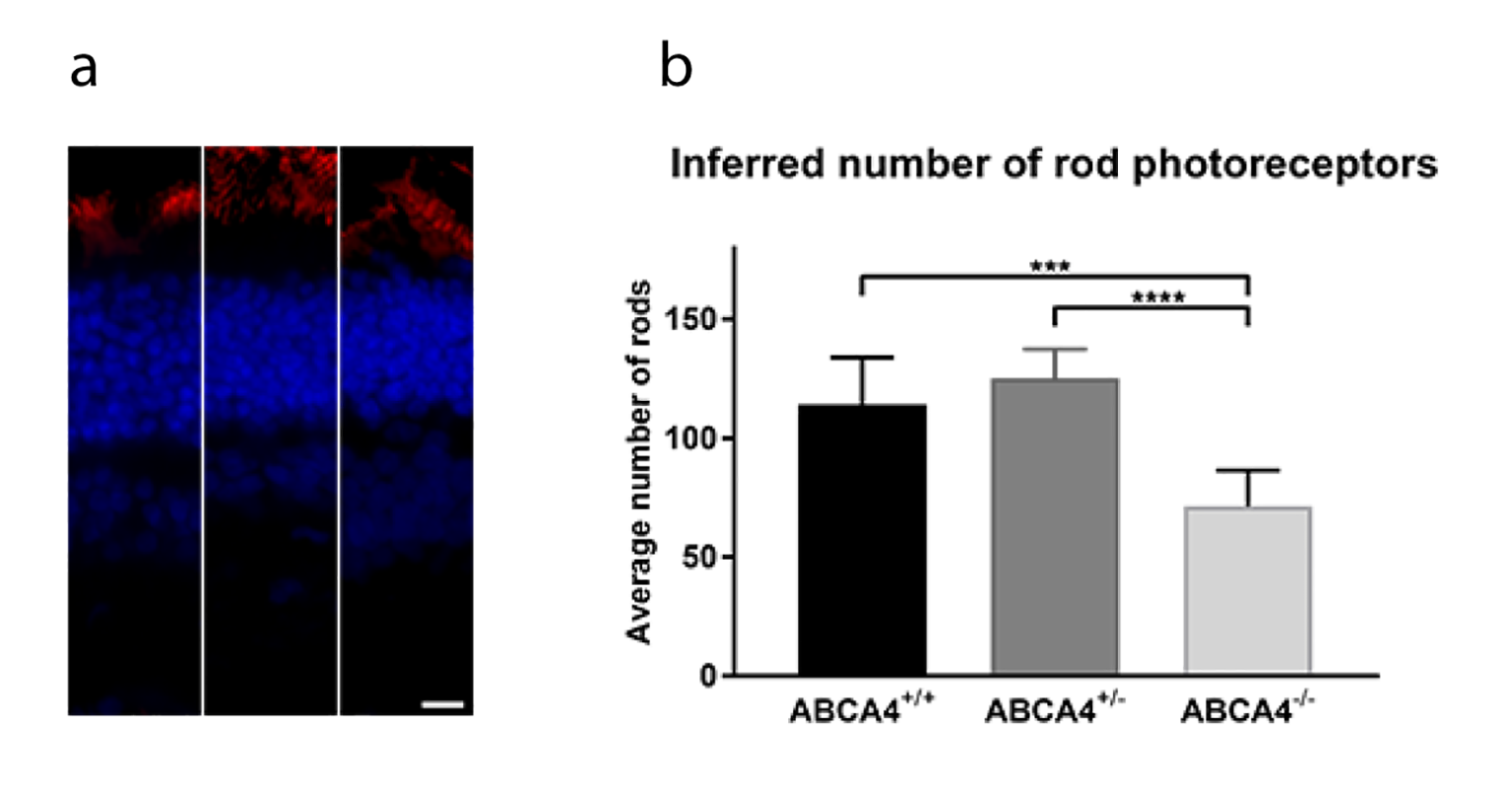

Supplement: S2 Fig — (A) Fluorescence micrographs showing rhodopsin expression (red) in the ABCA4+/+ (left), ABCA4+/- (middle), and ABCA4-/- (right) rod outer segments. Scale bar = 10 μm. (B) Inferred number of rod photoreceptors based on the number of nuclei in the outer nuclear layer and the number of cone photoreceptors within a given region of the retina. Cone photoreceptors were identified via PNA, which binds selectively to cone photoreceptors. Because there was only one individual per genotype, the statistics are valid for the technical replicates. ANOVA with Tukey’s post hoc test, n = 6; ***P < 0.001; ****P < 0.0001; mean ± S.D. (TIF) [file pgen.1007873.s002.tif]

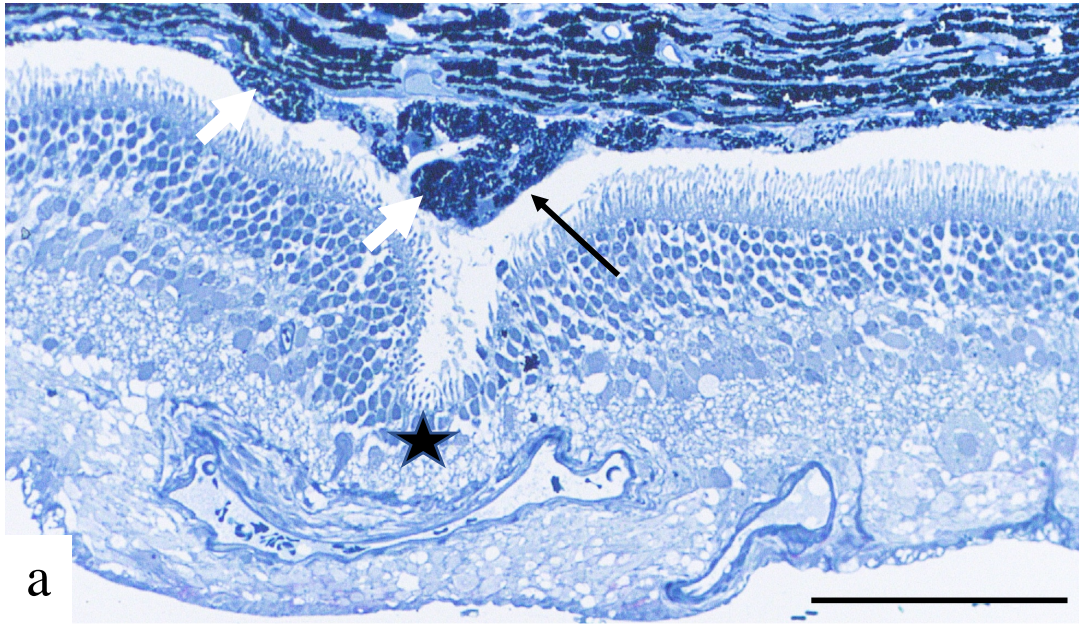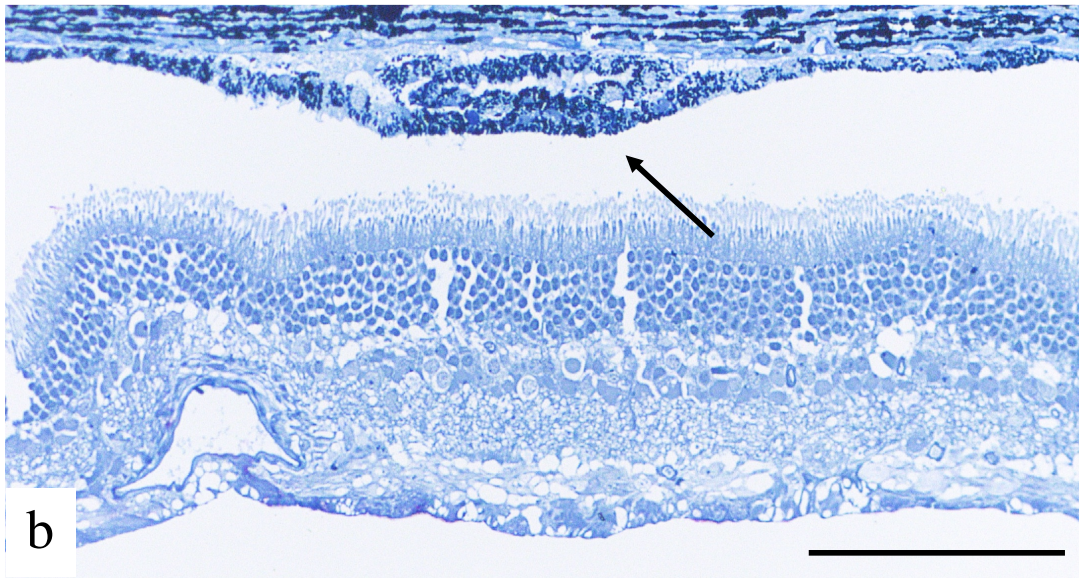

Supplement: S3 Fig — (A-B) Several focal regions of RPE hypertrophy (white arrows, A) as well as hyperplasia (black arrows, A, B), noted in two regions in the affected retina. Atrophy of overlying ONL and INL was noted over some (A; asterisk) of these regions. Lesions were focal (approximately 50–100 microns in diameter), intermittent and seen only in a section from nasal, nontapetal retina of the ABCA4-/- dog. All scale bars = 100 microns. (PDF) [file pgen.1007873.s003.pdf]

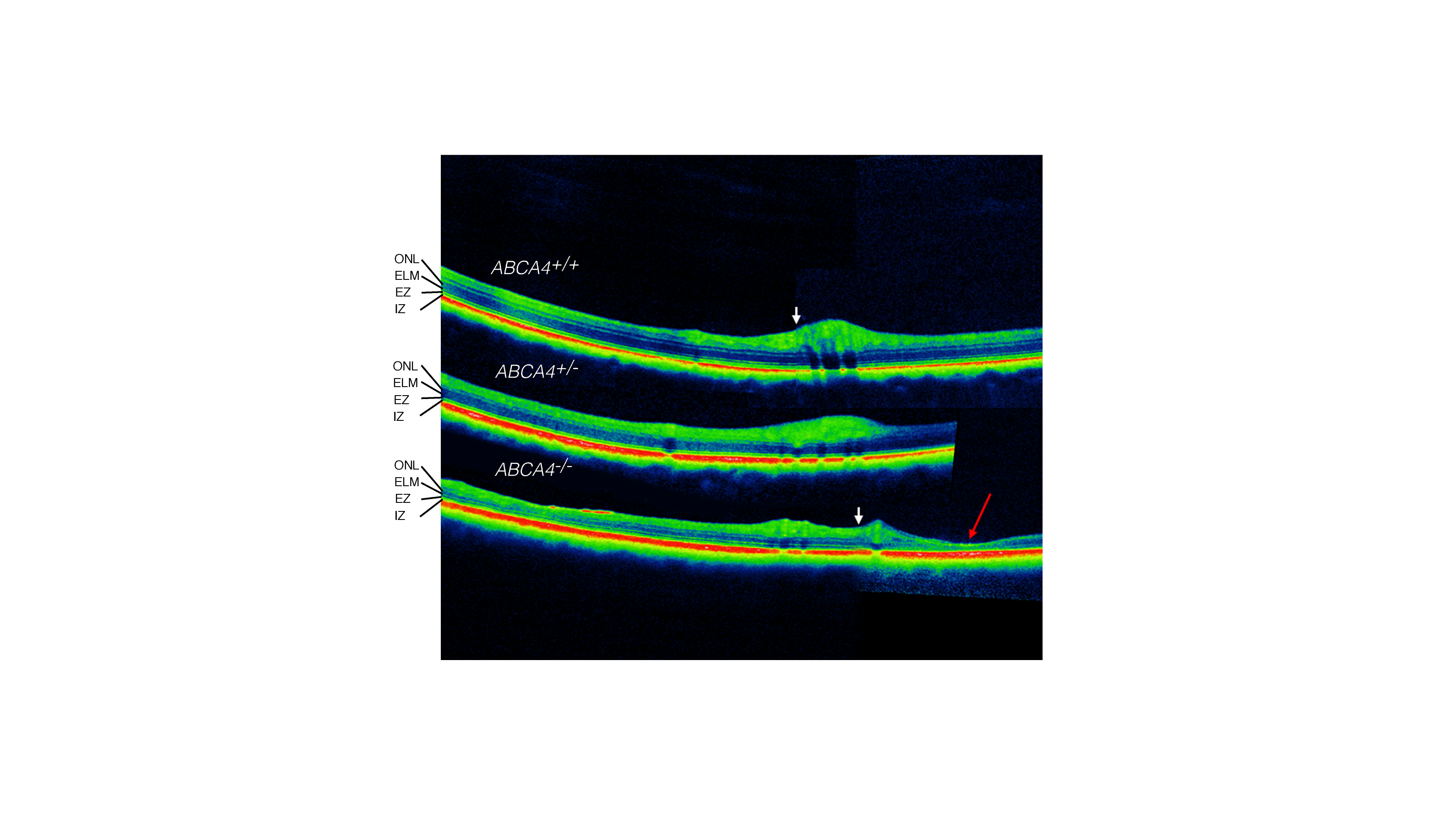

Supplement: S4 Fig — OCT scans from a 10-year old unaffected, wild-type dog (LAB22; top), a 12-year old heterozygous dog (LAB6; middle), and his affected littermate (LAB4; bottom). White arrows indicate where two images have been concatenated. A general thinning of ONL along the visual streak is visible in the affected retina compared to the wild-type and heterozygous retinas and included foci of severe retinal atrophy (red arrow). OCT = optical coherence tomography; ONL = outer nuclear layer; ELM = external limiting membrane; EZ = ellipsoid zone (inner-to-outer segment junction); IZ = outer segment-RPE interdigitation zone. (TIF) [file pgen.1007873.s004.tif]
